# Role of Adipokines Chemerin, Visfatin, and Omentin in Obesity and Their Inflammatory and Metabolic Implications

**DOI:** 10.3390/biomedicines13102321

**Published:** 2025-09-23

**Authors:** Wilson José S. Pedro, Flávio V. Barbosa Júnior, Fernanda N. B. R. Alves, Lenita V. Braga, Larissa R. Alves, João Pedro R. Afonso, Iranse O. Silva, Carlos Hassel M. Silva, Sergio Vencio, Paolo Capodaglio, Luis V. F. Oliveira, Wilson R. Freitas Júnior

**Affiliations:** 1 Graduate Program in Health Sciences at the Faculty of Medical Sciences of Santa Casa de São Paulo (PPGCS), 01224-001 Sao Paulo, Brazil; wilsonpedrosena@gmail.com (W.J.S.P.); keyciflavio@gmail.com (F.V.B.J.); drafernandabueno@yahoo.com (F.N.B.R.A.); lenita.braga@aluno.fcmsantacasasp.edu.br (L.V.B.); lari.ralves@gmail.com (L.R.A.); wilsonrfreitasjunior@gmail.com (W.R.F.J.); 2 Graduate Program in Human Movement and Rehabilitation (PPGMHR), Evangelical University of Goiás (UniEVANGELICA), 75083-515 Anapolis, Brazil; joaopedro180599@gmail.com (J.P.R.A.); iranseoliveira@hotmail.com (I.O.S.); carloshmendes@unievangelica.edu.br (C.H.M.S.); svencio@gmail.com (S.V.); 3 ICF–Institute of Pharmaceutical Sciences, 74935-530 Aparecida de Goiania, Brazil; 4 Orthopaedic Rehabilitation Unit and Research Lab for Biomechanics, Rehabilitation and Ergonomics, Ospedale San Giuseppe, Istituto Auxologico Italiano, IRCCS, 28824 Piancavallo di Oggebbio, Italy; p.capodaglio@auxologico.it; 5 Department of Surgical Sciences, Physical Medicine and Rehabilitation, University of Torino, 10126 Torino, Italy; 6 Physical and Rehabilitation Medicine Sector, Department of Biomedical, Surgical and Dental Sciences, Università Degli Studi di Milano (UNIMI), 20122 Milan, Italy

**Keywords:** obesity, adipokines, chemerin, omentin, visfatin, biomarkers, inflammation

## Abstract

Obesity is a multifactorial disease with endocrine, metabolic, and inflammatory underpinnings, leading to numerous comorbidities and increased mortality. This has driven research into adipose tissue’s role as an endocrine organ that secretes adipokines. This review critically analyzes three of these adipokines: chemerin, omentin-1, and visfatin. Chemerin and omentin-1 have well-defined roles as pro- and anti-inflammatory mediators, respectively. However, the function of visfatin remains controversial, with conflicting data regarding its role in glucose metabolism and inflammation. This conflicting evidence highlights an urgent need for standardized assays and population-specific studies to clarify its true function. We conclude that while chemerin and omentin-1 represent promising targets, the ambiguity surrounding visfatin limits its current clinical utility, and resolving these knowledge gaps is essential for developing effective biomarkers and therapies for obesity and its comorbidities.

## 1. Introduction

In its 2023 World Obesity Atlas, the World Obesity Federation presented a worrying obesity incidence in 2035 [1]. This trend is closely linked to global shifts in dietary patterns, where factors such as formal education and professional support are crucial in shaping healthy nutrition attitudes [2]. A global prevalence of overweight or obesity is projected at approximately 51% of the world’s population, representing an increase of 13% when compared with the 2020 data [3,4]. As a result of this incidence, the economic impact of obesity is estimated to reach a projected annual expenditure of approximately USD 4.32 trillion (approximately 3% of the global gross domestic product) if prevention and treatment measures are not effectively implemented [4,5]. In this context, obesity is a serious, chronic, and complex condition, directly related to several comorbidities such as cardiovascular disease, hypertension, type 2 diabetes (T2DM), and certain types of cancer [6,7,8]. Beyond these physiological consequences, obesity also presents significant social and healthcare challenges, including the prevalence of prejudice among health professionals, which can negatively impact patient care and treatment adherence [9].

A new understanding of obesity or excess fat has gained ground in recent decades, and body fat is being regarded as metabolically active, secretory, and responsive [10,11]. Adipose tissue is an endocrine organ that is dynamically involved in the regulation of cellular function and pathogenesis of diseases through a complex network of endocrine, paracrine, and autocrine signals that influence the response of many tissues, including the hypothalamus, pancreas, liver, skeletal muscle, kidneys, endothelium, and immune system [11,12]. These signals are mediated by proteins, such as hormones, growth factors, and cytokines, which are called adipokines [13]. Obesity alters the profile of these molecules, leading to an increase in pro-inflammatory adipokines such as leptin, chemerin, and visfatin, while levels of adiponectin and omentin, which exert counterbalancing anti-inflammatory effects, are typically reduced [11,13,14]. The secretion of these pro-inflammatory mediators is directly related to increase in M1 macrophages in adipose tissue, corroborating the idea of chronic inflammation related to obesity [15,16].

New inflammatory markers, such as omentin-1, visfatin, and chemerin, have demonstrated importance in the intricate regulatory network of obesity [17,18,19]. Chemerin is an adipokine that acts on autocrine and paracrine signals, thereby triggering adipocyte differentiation, modulating gene expression for glucose and lipid homeostasis, and stimulating lipolysis [20,21]. Studies have shown that circulating levels of chemerin are significantly higher in individuals with obesity [22]. In contrast, omentin, specifically its isoform 1, is an anti-inflammatory adipokine, expressed primarily in the vascular stroma of visceral adipose tissue (VAT) [23]. This protein plays a crucial role in the development of inflammatory diseases, a function supported by the observation that its circulating levels are typically decreased in these conditions. Its anti-inflammatory mechanisms include suppressing the expression of endothelial adhesion molecules such as VCAM-1 and ICAM-1 and promoting the polarization of macrophages toward an anti-inflammatory M2 phenotype, actions largely mediated by the inhibition of the NF-κB signaling pathway. These protective actions contribute to its beneficial effects on energy homeostasis, glucose metabolism, and the cardiovascular system [24,25,26]. Finally, visfatin, an enzyme that activates insulin and has insulinotropic effects, is an adipokine produced primarily by VAT [27]. It is involved in glucose metabolism and systemic inflammation, although there are conflicting studies regarding its relationship with serum levels, diabetes, and insulin resistance [28,29].

Due to new studies highlighting the importance of adipokines, it is important to understand the actual role of these cytokines in the body and how we can use them to treat obesity. Therefore, this review aimed to critically analyze the role of chemerin, visfatin, and omentin in the pathophysiology of obesity, observing their functions in the inflammatory and metabolic processes, thereby elucidating gaps that require further scientific effort.

## 2. Adipose Tissue as an Endocrine Organ in Obesity

Besides its primary function in energy storage, white adipose tissue (WAT) is now recognized as a highly active endocrine organ [30]. It is currently known that WAT secretes several cytokines, chemokines, and hormonal factors (adipokines) that regulate several processes, including energy expenditure, insulin sensitivity, feeding behavior, inflammation, and immunity [30]. WAT is organized into several depots in the body, including beneath the skin (subcutaneous), within a cavity (visceral), and in other small depots in organs. There are multiple physiological differences between VAT and subcutaneous adipose tissue. VAT adipocytes are more insulin-resistant, metabolically more active, and have greater lipolytic activity [30]. Furthermore, visceral fat accumulation is associated with an increased risk of developing T2DM and metabolic syndrome [31,32].

**Figure 1:**
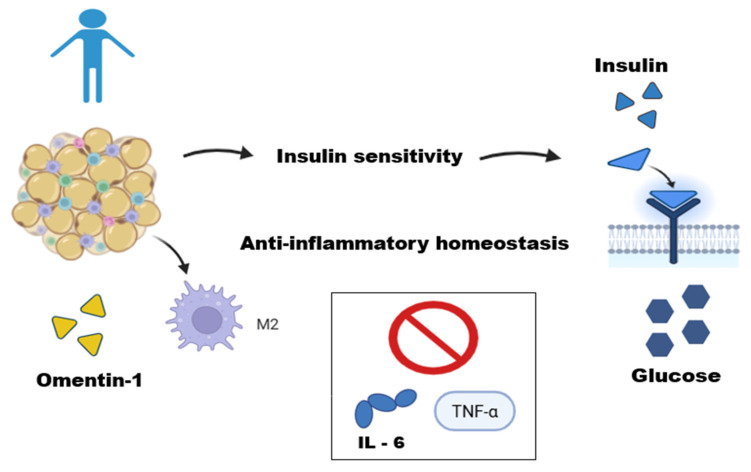
**Adipose tissue function in a lean individual.** This figure illustrates the role of adipose tissue in a lean individual as an active endocrine organ maintaining metabolic homeostasis. In these conditions, adipose tissue operates in equilibrium with immune cells, such as M2-type macrophages. The secretion of anti-inflammatory adipokines, like Omentin-1, helps regulate metabolism by improving insulin sensitivity, an effect mediated by potentiating the Akt signaling pathway in adipocytes and inhibiting inflammatory cytokines (IL-6 and TNF-α), thus contributing to anti-inflammatory homeostasis. This figure was made on Biorender.com.

In the WAT of lean individuals, homeostasis occurs through the regulation between adipocytes and immune cells [33]. These cells are predominantly represented by regulatory and immunosuppressive cells: type 2 macrophages (M2), regulatory T lymphocytes, T helper cells, natural killer T lymphocytes, and eosinophils [34,35,36,37,38]. M2 macrophages are uniformly distributed within adipose tissues, and they perform several physiological functions, including clearance of dead adipocytes, inhibition of progenitor adipocyte proliferation, and secretion of anti-inflammatory adipokines such as interleukins [39,40,41,42] (Figure 1). Furthermore, this group exhibits increased adiponectin secretion, which improves insulin sensitivity [41].

**Figure 2:**
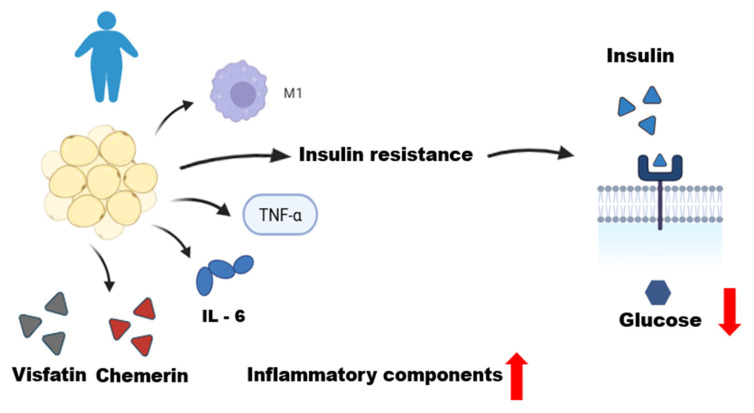
**Dysfunctional adipose tissue in an obese individual.** This figure illustrates the role of dysfunctional adipose tissue in an obese individual, characterized by a state of chronic low-grade inflammation. Qualitative and quantitative changes in adipose tissue, including adipocyte hypertrophy, lead to the infiltration of M1 macrophages and the production of inflammatory mediators. These include pro-inflammatory adipokines (chemerin and visfatin) and cytokines (TNF-α and IL-6). This inflammatory environment promotes systemic insulin resistance, resulting in decreased glucose uptake by the cells, as depicted by the reduced entry of glucose from the extracellular to the intracellular space. This figure was made on Biorender.com.

Obesity is recognized as a multifactorial chronic disease in which qualitative and quantitative changes in the histological structure of adipose tissue leads to consequent functional alterations that explain disease severity and development of its complications [43,44]. The primary cause of obesity is likely an altered association between the environment and genetic makeup of control systems, which underlies the regulation of energy metabolism and influences the bidirectional pathways of energy transfer between the environment and body [45]. Positive energy balance results in adipocyte expansion with complex changes in this equilibrium environment. Increased lipid storage results in adipocyte hypertrophy, hypoxia, and increased cell apoptosis [45,46]. This dysfunctional microenvironment promotes the secretion of pro-inflammatory cytokines by adipocytes, including tumor necrosis factor alpha (TNF-α), interleukins (IL)-6, and IL-8 [47,48,49] (Figure 2). Other chemokines produced by adipocytes or immune cells lead to infiltration and circulation of monocytes and other innate and adaptive immune cells within adipose tissue [46]. Increased monocytic infiltration results in a profound change in the adipose tissue environment, now with increased macrophage retention [33].

The distribution of cells is different in individuals with obesity. The observed inflammatory profile is characterized by the accumulation and infiltration of various immune cell populations in adipose tissue, with M1-type inflammatory macrophages predominating [50]. The adipose tissue of individuals with obesity releases pro-inflammatory adipokines such as TNF and IL-1β [50,51]. TNF is considered one of the main factors driving low-grade systemic inflammation in obesity [51]. Furthermore, adipose tissues, especially VATs, produce excess IL-6 in obesity [52]. The production of these pro-inflammatory cytokines contributes to metabolic inflammation and promotes insulin resistance [53,54,55].

Given the above, it is clear how adipose tissue plays important roles in local and systemic metabolic modulation, establishing a strong link between obesity and its comorbidities. Due to this complexity, the study of different metabolic profiles is fundamental for studies in this area [56]. The understanding of different metabolic profiles is still evolving. For instance, the phenotype of metabolically healthy obesity is estimated to affect 19% of the global population, while metabolic abnormalities can be found in 22% of lean individuals. This complexity is compounded by the lack of a universal scientific consensus on the definition of ‘metabolic health’, which makes the analysis and interpretation of data challenging [56]. Table 1 shows the main findings of this review on the role of adipokines in the pathophysiology of obesity.

This complexity is exemplified by the ongoing controversy surrounding the ‘metabolically healthy obesity’ (MHO) phenotype. Individuals with MHO are obese by BMI criteria, but they do not present a typical set of cardiometabolic abnormalities, such as insulin resistance, hypertension, and dyslipidemia [72]. The main point of debate is whether MHO is a stable, benign condition or merely a transient phase preceding the onset of metabolic disease. This controversy is compounded by the lack of standardization for MHO, making it difficult to compare results across studies [73].

Furthermore, longer-term studies suggest that individuals with MHO remain at higher risk of developing type 2 diabetes and cardiovascular events compared with lean, metabolically healthy individuals [74,75]. From an endocrine perspective, the adipokine profile in MHO is a key area of investigation, as these individuals often present intermediate levels of adipokines, such as adiponectin and leptin, compared with lean and metabolically unhealthy obese individuals. This profile suggests a less dysfunctional state of adipose tissue, which may explain their transiently protected phenotype [72,73,76].

This debate makes it clear that BMI alone cannot be considered a sufficient marker of cardiometabolic risk and highlights the need to understand underlying biological factors, such as adipokine secretion.

## 3. Chemerin: A Pro-Inflammatory Adipokine and Obesity

Chemerin, also known as tazarotene-induced gene 2 or retinoic acid receptor responder 2, is a chemotactic protein that is highly expressed in the liver and adipose tissues [57,58]. Most circulating chemerin is produced by the liver and found in an inactive form (pro-chemerin: 143 amino acids), which can be cleaved by extracellular proteases, including plasmin, elastase, and cathepsin G, into its active isoforms [77]. Isoforms 1 and 2 mediate the direct biological effects of chemerin by binding to high-affinity receptors such as chemokine-like receptor 1 and G protein-coupled receptor 1 [77,78].

These adipokines play an important role in adipocyte differentiation and function regulation by controlling the expression of the glucose transporter GLUT4, synthesis of triglycerides, and expression of leptin and adiponectin [79,80]. Human studies have found a positive correlation between their circulating levels, obesity, and body mass index (BMI) [59,60]. Other studies establish an association between their serum elevation and adipose tissue with metabolic syndrome [81]. To better understand the role of chimerin, we must evaluate its intracellular signaling pathways, the main pathway being initiated by its binding to the G-protein-coupled receptor, CMKLR1 (Chemokine-Like Receptor 1), which is abundantly expressed in adipocytes and immune cells, such as macrophages [61]. This interaction triggers distinct cascades that govern inflammatory and metabolic processes.

In the context of inflammation, CMKLR1 activation is directly linked to the nuclear factor kappa B (NF-κB) pathway. Chemerin signaling induces the phosphorylation and subsequent degradation of inhibitor of NF-κB (IκB), allowing NF-κB to translocate to the cell nucleus [82,83]. Once in the nucleus, it acts as a potent transcription factor, increasing the expression of an arsenal of pro-inflammatory genes. These include cytokines crucial in the pathophysiology of obesity, such as tumor necrosis factor alpha (TNF-α), interleukin-6 (IL-6), and interleukin-1β (IL-1β), thus perpetuating the chronic low-grade inflammatory state [82,83].

From a metabolic perspective, chemerin influences the AMP-activated protein kinase (AMPK) pathway, a master sensor of cellular energy status that promotes catabolic processes. In metabolically active tissues, such as adipose tissue and vascular endothelium, studies indicate that chemerin exerts an inhibitory effect on AMPK activity [84,85]. AMPK suppression results in decreased fatty acid oxidation and reduced glucose uptake, mechanisms that directly contribute to the development of insulin resistance and lipid accumulation. Therefore, by modulating these two central pathways (NF-κB and AMPK), chemerin functions as a crucial molecular bridge, connecting adipose tissue dysfunction with the systemic inflammation and metabolic dysregulation characteristic of obesity [61,84,85,86]. The chemokine-like receptor 1 expressed by macrophages, DCs, mast cells, neutrophils, and natural killer cells, also binds to chemerin, and its activation is involved in regulating immune cell recruitment to decrease an acute inflammatory response [86].

It is important to note, however, that while chemerin is predominantly pro-inflammatory in the chronic context of obesity, it can exhibit a dual role. In settings of acute inflammation, for instance, chemerin can contribute to the resolution phase by recruiting specific immune cell subsets that dampen the inflammatory response, highlighting the context-dependent nature of its biological function [58,61,80]. This complexity suggests that therapeutic interventions targeting the chemerin system must be highly specific to avoid unintended effects.

In vitro and in vivo studies have demonstrated chemerin involvement in energy balance and metabolism, with implications for obesity, diabetes, and metabolic syndrome [87,88]. According to one study, circulating chemerin levels were significantly higher in individuals with obesity individuals having a BMI > 25 kg/m^2^ compared with those with a BMI < 25 kg/m^2^. In humans, circulating chemerin levels have been strongly associated with multiple components of metabolic syndrome, including BMI, triglycerides, and high-density lipoprotein cholesterol, as well as with markers of systemic inflammation such as C-reactive protein, IL-6, and TNF-α [89].

Due to the strong association of chemerin with metabolic diseases, many studies are evaluating its receptor, CMKLR1, as a potential therapeutic target. A preclinical study with obese diabetic mice has shown that the CMKLR1 antagonist, CCX832, yielded promising results [90]. Treatment with this antagonist led to improvements in important metabolic parameters, including a reduction in body weight, insulin, and glucose levels, as well as the restoration of vascular response to insulin [90]. Furthermore, blocking the chemerin receptor has also demonstrated benefits in related comorbidities, such as the attenuation of renal oxidative stress in diabetic mice [91]. Despite these interesting preclinical findings, the clinical development of chemerin-specific interventions for obesity and type 2 diabetes remains in its early stages. There is a notable lack of published data from late-stage clinical trials focusing on these specific conditions, indicating that more research is necessary to validate the safety and efficacy of this therapeutic approach in humans.

## 4. Visfatin: A Complex Profile in Obesity and Inflammation

Visfatin, a cytokine originating from VAT, has a complex profile in obesity and inflammation. It was initially identified as a lymphokine that acts as a growth and differentiation factor for B lymphocyte precursors [62]. Also known as pre-B cell colony-enhancing factor and later as nicotinamide phosphoribosyl transferase (NAMPT), visfatin has been associated with an insulin-mimetic effect by binding and activating the insulin receptor. Its tissue expression and secreted plasma levels increase parallel to obesity, making it an interesting area for future studies [62,92].

Studies related to visfatin have shown a relationship between bioenergetic metabolism and the nicotinamide-adenine dinucleotide oxidation (NAD+) pathway, suggesting a potential role for NAD-dependent enzymes in regulating the production of pro-inflammatory cytokines [92,93]. Visfatin/NAMPT is produced and released predominantly by adipose tissue-derived macrophages. Its association with inflammatory markers, such as the upregulation of IL-6, IL-1b, and TNF-α in human monocytes, suggests inflammatory properties [93]. The inflammatory response in adipose tissue is also linked to selective hypoxia in hypertrophied adipocytes, and studies indicate that circulating mononuclear cells in patients with obesity are in a pro-inflammatory state [94,95].

However, the relationship between visfatin and obesity is still unclear. The controversy surrounding visfatin was amplified by significant historical events. The seminal study by Fukuhara, which initially generated great enthusiasm by describing it as an adipokine with insulin-mimetic effects, was later retracted, fueling an intense debate in the scientific community that persists to this day [27]. Although visfatin mRNA expression in VAT correlates with obesity, its circulating levels show variable associations [93]. Recent evidence suggests that when the visfatin gene is expressed in adipose tissues, it does not necessarily correlate with its circulating levels, which may explain the inconsistent results. Furthermore, a positive correlation can be observed between plasma visfatin levels and visceral fat in humans [96]. Other studies reiterate this conflicting association of plasma levels with BMI and other anthropometric measurements [62,92]. Visfatin expression appears to be locally regulated in the VAT of individuals with obesity, where it is secreted by activated macrophages to act in an autocrine manner within a fat depot [96]. This increased local production, especially in cases of intra-abdominal obesity, may be a crucial factor in changing the properties of visfatin [96].

Furthermore, the relationship between visfatin with insulin resistance and T2DM also remains a topic of debate. The literature presents conflicting results regarding visfatin levels in different metabolic conditions. For example, some studies found that serum visfatin levels were similar in patients with T2DM, glucose intolerance, and those with normal glucose tolerance [63,64]. Furthermore, the relationship between visfatin with insulin resistance and T2DM also remains a topic of debate, with studies presenting conflicting results [65]. On one hand, a line of research demonstrates a positive correlation, where studies using the ELISA method have found significantly higher visfatin levels in populations with clear metabolic dysfunction—such as patients with obesity and metabolic syndrome or in psoriasis patients who also present with metabolic syndrome—when compared to healthy controls [62,66,97].

On the other hand, a substantial body of evidence points to a more complex association. For example, a systematic review and meta-analysis highlighted elevated visfatin levels in non-obese women with Polycystic Ovary Syndrome (PCOS). This finding, echoed in other studies on non-obese women with PCOS and patients with type 2 diabetes, suggests a link to the pathology that can be independent of obesity [98,99,100]. To systematically organize these divergent findings, Table 2 provides a comparative summary of these representative studies. These inconsistencies in the literature may be due to differences in study populations, including age, sex, and associated conditions [101,102].

The regulatory pattern of visfatin is complex. For instance, intravenous glucose infusion elevates its levels, a response that could be mechanistically linked to its role as the enzyme NAMPT (nicotinamide phosphoribosyltransferase). As NAMPT is the rate-limiting enzyme in the NAD+ salvage pathway, an acute glucose load increases the cellular demand for NAD+ to sustain glycolysis, potentially stimulating visfatin/NAMPT secretion to support this metabolic need [103]. Conversely, oral carbohydrate ingestion may reduce its levels, indicating that it may not follow the conventional adipokine profile [104,105]. An interesting hypothesis is that elevated visfatin levels could be a regulatory response to maintain glucose homeostasis, but an excessive increase may contribute to chronic inflammation, which in turn may lead to insulin resistance and T2DM [63,105,106].

Another factor that may be relevant to understanding this inconsistency in the literature is the variability in the assay methods used to measure visfatin levels and the fact that circulating levels may not reflect its local activity in visceral adipose tissue. For example, some studies that reported a positive correlation with obesity were conducted in specific populations with metabolic syndrome, while studies that found no significant association included more heterogeneous cohorts [23,29,30].

## 5. Omentin-1: An Anti-Inflammatory Adipokine and Its Role in Obesity

Omentin-1 is an adipokine, and the isoform 1 of omentin is its main circulating form in the body [107]. Its expression occurs in the vascular stroma of VAT, in greater quantity than in subcutaneous adipose tissue, and also shows high expression in epicardial adipose tissue [108]. Omentin-1 has emerged as an important key in the complex interaction between adipose tissues and the body’s physiological processes [67]. Reduced circulating levels of omentin-1 have been consistently associated with obesity, insulin resistance, and cardiovascular dysfunction [68,109]. In fact, there is a negative correlation between serum concentrations of omentin-1 and BMI, the insulin resistance index, leptin, blood glucose, and Homa B, as well as pro-inflammatory cytokines such as TNF-α, L-1β, and IL-6 [69,110,111]. Several factors, including obesity, insulin sensitivity, inflammation, genetic factors, and hormones, such as adiponectin and insulin, can affect the production and secretion of omentin-1 [25,112].

Omentin-1 may exert its metabolic and anti-inflammatory effects in a linked manner. This adipokine inhibits pro-inflammatory cytokines, such as TNF, IL-6, IL-1β, and other cytokines, such as IL-8 and CCL2, while increasing the secretion of anti-inflammatory adipokines such as IL-10 and adiponectin [70,71,94]. Furthermore, omentin-1 inhibits oxidative stress in the endoplasmic reticulum and mitochondrial dysfunction [113]. Omentin-1 has gained attention due to its potential significance in vascular function and its role in glucose and lipid metabolism and insulin sensitivity [94]. Omentin-1 levels have been shown to decrease in dysmetabolic conditions, being downregulated according to glucose-insulin levels [114]. However, basal glucose transport is not stimulated independently, as it generates an improvement in insulin-mediated glucose uptake in adipose tissues, presenting a mode of action similar to that of adiponectin [70]. Some factors, such as physical exercise, aerobic exercise, and weight loss, are linked to high levels of omentin-1 [113].

Vascular function improves with positive effects due to omentin-1, making it an important target for studies. It promotes vasodilation, thereby exerting anti-inflammatory and potentially anti-atherosclerotic actions [115,116]. The mechanism of vasodilation involves stimulating nitric oxide production by endothelial cells, which relaxes vascular smooth muscle, thereby reducing vascular resistance and improving blood flow [116]. A recent study showed that omentin-1 plays important roles in endothelial cells through anti-inflammatory, antioxidant, and anti-apoptosis activities, preventing endothelial dysfunction and contributing to cardioprotection [116].

Omentin-1 may play an important role in fat breakdown (lipolysis) and lipid storage in adipose tissues [117]. The interactions between this adipokine and other hormones, such as insulin and adiponectin, contribute to the complex signaling network that controls lipid and glucose metabolism [118]. Given its important clinical roles, therapies targeting increased omentin-1 levels could be used for the treatment of conditions such as atherosclerosis, hypertension, and T2DM [119,120]. Omentin-1 should be explored as a potential biomarker for certain vascular and metabolic conditions. The potential of omentin-1 as a clinical biomarker has been proposed by several authors. Recent studies using ROC curve analysis have proposed specific diagnostic cut-off values. For example, in a study with obese patients, a cut-off value of ≤ 372.45 ng/mL was suggested to identify individuals with metabolic syndrome [70]. In another study focused on patients with hypertension, a lower cut-off value of ≤ 62.20 ng/mL was identified to predict the development of the syndrome [121]. Notably, these proposed cut-off values vary considerably across studies. This variability is likely due to differences in the cohorts studied (e.g., ethnicity, underlying clinical conditions), the assay methods used, and the specific definition of the condition being diagnosed. Therefore, while several studies have established these cut-off points, further large-scale validation studies are necessary to establish standardized, clinically applicable cut-off values before omentin-1 can be widely adopted as a diagnostic biomarker.

## 6. Conclusions

Chemerin and omentin-1 have well-defined roles as pro- and anti-inflammatory adipokines, whereas visfatin remains controversial due to conflicting data. Based on this review, we reinforce that chemerin is a pro-inflammatory adipokine strongly associated with obesity, insulin resistance, and metabolic syndrome. However, visfatin has shown inconsistent results in the literature, indicating a complex systemic or local role in adipose tissues. Finally, omentin-1 stands out as an anti-inflammatory adipokine; some low circulating levels are associated with obesity and its comorbidities. Its beneficial role in insulin sensitivity and vascular function makes it a promising therapeutic target.

Despite the progress made, a complete understanding of adipokine mechanisms still requires further investigation. Future research should focus on elucidating specific downstream signaling pathways, such as the role of chemerin in activating the CMKLR1 receptor, which modulates inflammatory responses through the NF-κB pathway, or how omentin-1 enhances insulin sensitivity via the Akt/eNOS signaling cascade. Furthermore, the complex role of visfatin as the enzyme NAMPT in regulating NAD+ metabolism offers a promising avenue for understanding its dual role in metabolic homeostasis and inflammation. Complementing this mechanistic focus, future studies should include genetic analyses and investigations in diverse populations and environments. This integrated approach is essential for fully uncovering the therapeutic potential of these molecules and validating their use as reliable biomarkers in the treatment of obesity and its associated diseases, such as T2DM and cardiovascular disease.

## Figures and Tables

**Table 1:** Role of adipokines chemerin, visfatin, and omentin-1 in the pathophysiology of obesity.

Adipokine	Classification	Tissue Of Origin/Main Expression	Association With Obesity	Main Functions
Chemerin Refs. [20,21,57,58,59,60,61]	Pro-inflammatory	Liver and adipose tissue	Significantly higher circulating levels in obese individuals	It acts on adipocyte differentiation, modulates glucose and lipid homeostasis, and stimulates lipolysis. It recruits plasmacytoid dendritic cells, initiating an innate immune response.
Visfatin Refs. [62,63,64,65]	Complex profile	Visceral adipose tissue and adipose-derived macrophages	Variable and conflicting association with obesity. Expression in visceral adipose tissue (VAT) correlates with obesity, but not necessarily with circulating levels.	It activates insulin and has insulinotropic effects by binding to and activating the insulin receptor. Involved in glucose metabolism and systemic inflammation.
Omentin-1 Refs. [66,67,68,69,70,71]	Anti-inflammatory	Vascular stroma of visceral adipose tissue	Reduced circulating levels in obese individuals with poor glycemic regulation	Circulating levels of omentin-1 are reduced in obese individuals, suggesting a role in insulin resistance, diabetes, obesity, and metabolic syndrome. Omentin-1 inhibits pro-inflammatory cytokines (such as TNF, IL-6, and IL-1) and increases the secretion of anti-inflammatory adipokines (such as IL-10 and adiponectin).

**Table 2:** Summary of representative studies on the association between visfatin and obesity.

Category	Study	Population Characteristics	Test Method	SS
Positive Correlation	Ugur, K. et al., 2022 [62]	Patients with obesity and metabolic syndrome vs. healthy controls.	ELISA	Significantly higher visfatin levels in patients with obesity and metabolic syndrome, suggesting its role as a biomarker.
Positive Correlation	Sherly A A et al., 2025 [66]	Case–control study with patients with metabolic syndrome vs. healthy controls.	ELISA	Significantly altered visfatin levels in patients with metabolic syndrome, a condition strongly associated with obesity.
Positive Correlation	Dağdelen D et al., 2020 [97]	Patients with psoriasis, with and without Metabolic Syndrome.	ELISA	Higher visfatin levels in patients with Metabolic Syndrome, suggesting a link with metabolic dysfunction.
No correlation/Variable	Chen J et al., 2023 [98]	Meta-analysis of studies including non-obese women with Polycystic Ovary Syndrome (PCOS).	Meta-análise	Higher visfatin levels in non-obese women with PCOS compared with controls, indicating an association with pathology independent of obesity.
No correlation/Variable	Kärberg, K. et al., 2023 [99]	Patients with type 2 diabetes.	ELISA	No direct association found between visfatin and atherosclerosis; results were influenced by the use of cardiovascular medications.
No correlation/Variable	Ali and Nori, 2022 [100]	Non-obese women with polycystic ovary syndrome (PCOS) vs. healthy controls.	ELISA	No significant correlation was found between visfatin levels and BMI. Levels were elevated in PCOS, regardless of obesity.

## Data Availability

The data presented in this study will be made available upon request to the corresponding author due to ethical restrictions.

## Abbreviations

The following abbreviations are used in this manuscript:

T2DM	Type 2 diabetes mellitus
VAT	visceral adipose tissue
WAT	White adipose tissue
TNF-α	Tumor necrosis factor alpha
IL	Interleukin
NAD	Nicotinamide-adenine dinucleotide
NAMPT	Nicotinamide phosphoribosyl transferase
BMI	Body mass index
